# Editorial for the Special Issue “Microbial Nonribosomal Synthesis of Secondary Metabolites”

**DOI:** 10.3390/microorganisms10051064

**Published:** 2022-05-21

**Authors:** Stéphane Cociancich, Valérie Leclère

**Affiliations:** 1 CIRAD, UMR PHIM, F-34398 Montpellier, France; 2 PHIM, Université Montpellier, CIRAD, INRAE, Institut Agro, IRD, F-34398 Montpellier, France; 3 Université de Lille, Université de Liège, UMRT 1158 BioEcoAgro, Métabolites Secondaires d’Origine Microbienne, Institut Charles Viollette, F-59000 Lille, France; valerie.leclere@univ-lille.fr

Microbial secondary metabolites are natural products that display various therapeutical or agrochemical relevant activities (e.g., antibiotics, antifungal, or antiproliferative agents, siderophores, or toxins). The identification of new secondary metabolites is of major interest, as their activities give them a prime placement in the “One-Health” worldwide concept triad that connects healthy people, healthy animals, and healthy environments. Bacteria and fungi produce many of these compounds as a courtesy of nonribosomal biosynthetic enzymatic pathways, which involve multimodular megaenzymes named nonribosomal peptide synthetases (NRPSs). This Special Issue includes a review highlighting recent progress in understanding the complexity of the mechanisms of nonribosomal synthesis [1], and five research articles describing the successful discovery of new nonribosomal peptides [2,3,4,5], their structural characterization [3,5], and their relationship with the taxonomic position of the producing strains [6].

NRPSs exhibit a fascinating thiotemplate-based characteristic architecture that permits the specific assembly of proteinogenic or nonproteinogenic monomers (e.g., amino acids, hydroxyacids, acyl-thioesters, fatty acids, chromophores). NRPSs are organized into modules, each of which is responsible for the specific incorporation of a given monomer into the growing peptidic chain. The mechanism leading to the assembly of the monomers to build up the peptide generally follows canonical rules using the core enzymatic domains referred to as A (adenylation) domain, T (thiolation) domain, C (condensation) domain, and a TE (thioesterase) domain ending the assembly line. However, the constantly expanding literature presents many examples of NRPSs exhibiting very rare domains and/or noncanonical organizations of domains and modules, thus revealing amazing strategies developed by microorganisms to synthesize nonribosomal peptides [1].

Genes encoding these huge synthetases are often clustered with other gene-encoding enzymes required for the synthesis of rare monomers, regulation, immunity, transport, or for tailoring (among others) proteins, leading to the concept of biosynthetic genes clusters (BGCs) being responsible for the biosynthesis, structural modifications, regulation, and transport of the corresponding secondary metabolites. For decades, new secondary metabolites were discovered through screenings of biological activities, but recently, increasing rediscoveries of already-known compounds proved that this strategy has become less efficient. However, the current exponential increase in available genomic sequences (including sequences from thousands of complete microbial genomes) suddenly gives access to a considerable amount of information which unravels the potential of microorganisms to produce secondary metabolites exhibiting new structural scaffolds, and therefore, new biological activities. This paved the way for another strategy to seek new metabolites based on in silico analyses of microbial genomes. This strategy is also known as genome mining. For the identification of new nonribosomal peptides, genomes can be mined for either BGCs containing genes encoding NRPSs with modular organization (including specific enzymatic domains), or genes encoding enzymes involved in biosynthesis of rare monomers. A major advantage of following a genome mining strategy rather than bioassay-based screenings is the detection of poorly expressed or unexpressed BGCs. Indeed, the mining over hundreds of genomes together with wet lab production assays reveals that most of the BGCs may remain silent under usual laboratory culture conditions. The actual challenge is to exploit this metabolic potential using techniques to awaken these silent/cryptic BGCs or using production in heterologous hosts. With these approaches, the metabolic potential appears vastly superior to previous estimates based on compounds isolated only on the basis of their biological activity.

In silico analyses of microbial genomes have benefited from recent efforts to develop bioinformatic tools for automatized detection of BGCs, determination of NRPS organization in modules and domains, and prediction of monomers incorporated by the various A domains. This resulted in the design of the chemical structures of the metabolites expected to be produced. These bioinformatic tools include online software such as antiSMASH (http://antismash.secondarymetabolites.org) or NapDOS (https://npdomainseeker.sdsc.edu/) as well as NRPS databases such as Mibig (https://mibig.secondarymetabolites.org) or Norine, the unique resource on nonribosomal peptides (http://norine.univ-lille.fr/norine/). Nevertheless, these bioinformatic resources are currently unable to handle NRPS pathways diverging from the canonical rules.

Aiming at presenting current and future directions in understanding the nonribosomal biosynthesis of microbial secondary metabolites, this Special Issue includes five research articles and one review which all show that the discovery and characterization of new nonribosomal secondary metabolites must consider numerous BGCs organizations and biosynthetic pathways beyond the canonical rules described above.

In their review, Duban et al. [1] discuss examples of secondary metabolites biosynthesized through pathways diverging from the canonical colinear mode, and hence involving NRPSs displaying either very rare domains and/or a noncanonical organization of modules and domains. This long (although probably inexhaustive) list of examples tells us that it is necessary to enlarge our vision of the nonribosomal peptide synthesis. Indeed, the more we learn about these biosynthesis pathways, the easier it will be to identify new natural products, especially through genome mining. Nevertheless, this very popular in silico strategy used for the discovery of potential new products of interest is pointless if not supported by the actual production, isolation, structural characterization, and determination of the mode of action of the corresponding natural products.

Although nonribosomal peptide synthesis is widespread within the bacterial and fungal kingdoms, all five research articles from this Special Issue provide examples of secondary metabolites produced by strains belonging to *Streptomyces* spp. *Streptomyces* are the source of thousands of previously described secondary metabolites and, as such, are often designed as metabolic factories. This shows that a genome mining approach may lead to the discovery of new metabolites from a genus, which is a reservoir for a very large number of compounds of interest. Interestingly, four out the five research articles describe nonribosomal peptide synthesis mechanisms that mainly work out of the canonical rules. The in silico analysis of nonribosomal BGCs, together with heterologous expression, represents a successful strategy that resulted in the discovery of BE-18257 antibiotics and pentaminomycins [2], cyclic depsibosamycins [3], and bonsecamin [4].

A surprising BGC organization is highlighted in the work of Roman-Hurtado et al. [2], which reports the presence of two NRPS-encoding genes within the same BGC. These genes are involved in the biosynthesis of two unrelated secondary metabolites (pentaminomycins and BE-18257 peptides) that are both cyclic pentapeptides but with very different structures. Interestingly, most of the enzymes encoded by the other genes within the BGC are shared during the biosynthesis of the respective products. Moreover, no TE domain was found in any of the NRPSs present within the BGC. Consequently, the release and macrocyclization of pentaminomycins and BE-18257 peptides is most likely performed by a stand-alone penicillin binding domain-type TE.

In their article, Stierhof et al. [3] deciphered the biosynthesis of depsibosamycins, which are cyclic octapeptides differing from their already-known linear counterparts (bosamycins) due to a four-amino acid ring formed by a side-chain-to-tail lactonization of serine and glycine. The NRPSs encoded by the BGC of depsibosamycins do possess a rather canonical organization, with the remarkable exception of a stand-alone NRPS module missing the C domain but possessing a CytP450 domain, allowing the incorporation of a subsequently methylated 5-OH-Tyr moiety.

Lasch et al. [4] describe the biosynthesis of the cyclic pentapeptides named bonsecamins. Bonsecamins are produced in a linear form and undergo further intramolecular dehydrative cyclization through the action of the products of two out of the seven genes forming the BGC. Bonsecamins NRPSs are formed by only three modules (one of them organized in a noncanonical way), which do not fit with the colinear canonical organization of NRPSs synthesizing pentapeptides. Remarkably, one of these modules incorporates a tripeptide produced by a NRPS-independent mechanism involving a putative alanine ligase. Such organization renders it difficult to make any prediction solely based on bioinformatic tools.

Another method of genome mining is to search for genes encoding rare amino acid biosynthesis enzymes and not to search directly for BGCs containing NRPS. Following this strategy, Horbal et al. [5] unraveled the biosynthetic pathway for the cyclohexapeptide cyclofaulknamycin that contains the rare D-capreomycidine monomer. The biosynthesis of this rare amino acid is partially performed by genes within the BGC, but it also requires the action of a pyridoxal-phosphate-dependent aminotransferase encoded by a gene located elsewhere in the genome. Interestingly, the NRPSs involved in cyclofaulknamycin biosynthesis do not contain any TE domain. Although a stand-alone type-II TE domain is present within the BGC, it is predicted to have a proofreading activity, whereas the release and cyclization of the cyclic hexapeptide would rather rely on an α/β hydrolase encoded within the BGC.

Finally, the fifth research article from this Special Issue is authored by Komaki et al. [6], who performed genome mining within *Streptomyces hygroscopicus* strains. More precisely, these authors analyzed the conserved BGC for nyuzenamide, a bicyclic peptide exhibiting antifungal and cytotoxic activities, in silico. Hence, they confirmed the correlation between taxonomic classifications of *S. hygroscopicus* strains obtained by 16S rDNA comparisons and by in silico analysis of nyuzenamide BGCs. As a result, strains of *S. hygroscopicus* were reclassified into two subspecies named *S. sporocinereus* and S. *hygroscopicus* subsp. *hygroscopicus*, respectively.

In conclusion, the original articles and review published in this Special Issue are interesting illustrations of the importance of improving and increasing our knowledge of nonribosomal peptide synthesis. This is reinforced by nonribosomal pathways’ complexity in comparison to canonical pathways, which are already complicated in their own right. Each new peptide discovered through bioactivity screening or genome mining is of considerable importance to feed data regarding diversity of biosynthetic pathways and to improve the accuracy of bioinformatics tools.
